# Speckle-tracking global longitudinal strain predicts death and cardiovascular events in patients with systemic sclerosis

**DOI:** 10.1093/ehjopen/oeae023

**Published:** 2024-04-03

**Authors:** Giulia Stronati, Federico Guerra, Devis Benfaremo, Cristina Dichiara, Federico Paolini, Gianmarco Bastianoni, Leonardo Brugiatelli, Michele Alfieri, Paolo Compagnucci, Antonio Dello Russo, Gianluca Moroncini

**Affiliations:** Cardiology and Arrhythmology Clinic, Marche Polytechnic University, Marche University Hospital, Via Conca 71, Ancona 60126, Italy; Cardiology and Arrhythmology Clinic, Marche Polytechnic University, Marche University Hospital, Via Conca 71, Ancona 60126, Italy; Clinica Medica, Marche Polytechnic University, Marche University Hospital, Via Conca 71, Ancona 60126, Italy; Internal Medicine Residency Programme, Marche Polytechnic University, Via Conca 71, Ancona 60126, Italy; Cardiology and Arrhythmology Clinic, Marche Polytechnic University, Marche University Hospital, Via Conca 71, Ancona 60126, Italy; Cardiology and Arrhythmology Clinic, Marche Polytechnic University, Marche University Hospital, Via Conca 71, Ancona 60126, Italy; Cardiology and Arrhythmology Clinic, Marche Polytechnic University, Marche University Hospital, Via Conca 71, Ancona 60126, Italy; Cardiology and Arrhythmology Clinic, Marche Polytechnic University, Marche University Hospital, Via Conca 71, Ancona 60126, Italy; Cardiology and Arrhythmology Clinic, Marche Polytechnic University, Marche University Hospital, Via Conca 71, Ancona 60126, Italy; Cardiology and Arrhythmology Clinic, Marche Polytechnic University, Marche University Hospital, Via Conca 71, Ancona 60126, Italy; Clinica Medica, Marche Polytechnic University, Marche University Hospital, Via Conca 71, Ancona 60126, Italy

**Keywords:** Echocardiography, Global longitudinal strain, Speckle-tracking, Cardiomyopathy, Systemic sclerosis, Scleroderma

## Abstract

**Aims:**

Albeit often asymptomatic, heart involvement in systemic sclerosis (SSc) represents a negative prognostic factor, accounting for nearly one-fourth of all deaths. Global longitudinal strain (GLS) is accurate in detecting heart involvement in patients with SSc and no overt cardiac disease and allows early detection and longitudinal monitoring, but its association with clinical endpoints has not been tested so far. The primary outcome was the association between left and right GLS and mortality for all causes. The secondary outcome was the association between left and right GLS and hospitalizations.

**Methods and results:**

A prospective longitudinal study enrolling all consecutive patients with SSc without structural heart disease or previous cardiovascular event.

A total of 164 patients were enrolled, of whom 19 (11.5%) died during follow-up and 48 (29.3%) were hospitalized. Both left (LV) and right ventricle (RV) GLS at enrolment were independently associated with an increased risk of death for all causes and hospitalizations. Patients with biventricular GLS impairment, respectively, had a 4.2-, 4.9-, and 13.9-fold increased risk of death when compared with patients with only LV, only RV, or no impairment (*P* < 0.001). The incidence of hospitalization in patients with biventricular GLS impairment was nearly four times higher when compared with patients with only LV or only RV impairment, and nine times higher when compared with normal biventricular GLS (*P* < 0.001).

**Conclusion:**

Biventricular GLS is associated with an increased risk of death and hospitalization in patients with SSc during a median of 3-year follow-up, acting as a reliable and accurate prognostic tool in everyday practice.

## Graphical Abstract

**Graphical Abstract:**
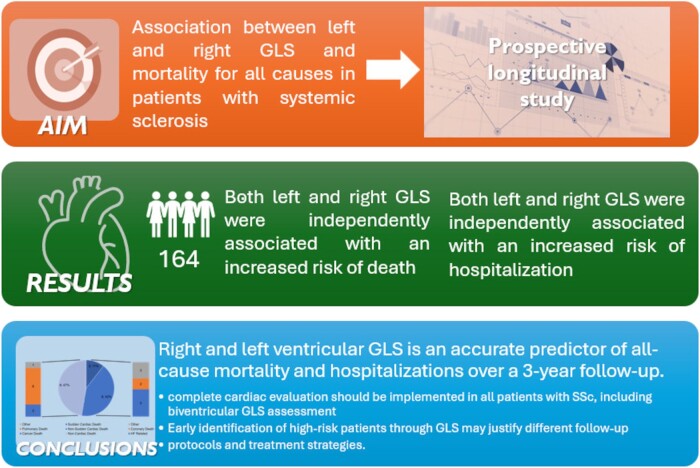
All-cause death in patients with systemic sclerosis and time to death, according to left and right global longitudinal strain. GLS, global longitudinal strain; SSc, systemic sclerosis.

## Introduction

Systemic sclerosis (SSc) is a chronic immune-mediated disease of unknown aetiology characterized by diffuse microvascular damage and significant accumulation of extracellular matrix, resulting in fibrosis of the skin and internal organs.^1,2^ Systemic sclerosis–related cardiomyopathy, which is also known as SSc-primary heart involvement,^3^ is a major complication in SSc and, albeit often asymptomatic, represents a negative prognostic factor.^4,5^ The prevalence of cardiac involvement varies between 15 and 35%^6^ and accounts for 26% of death causes in patients with SSc, mainly due to heart failure (HF) and arrhythmias.^6^

Current literature^7–9^ suggests that the pathophysiology of heart involvement in SSc is secondary to microvascular dysfunction and subsequent hypoxia, ischaemia, and fibrosis. The exact pathogenesis is not fully understood, but it is thought to involve a complex interplay among endothelial dysfunction, immune dysregulation, and fibrosis. The resulting damage to the heart can lead to various cardiac abnormalities that are predominantly attributable to SSc rather than other causes or complications.^10,11^ These abnormalities may be subclinical and must be confirmed through diagnostic investigation.^3^

To date, speckle-tracking-derived global longitudinal strain (GLS) has been proved to be a cost-effective and sensitive tool in the detection of left (LV) and right ventricle (RV) dysfunction in patients with SSc and no overt cardiac disease,^12–14^ with a recent meta-analysis showing that left ventricular GLS, circumferential strain, and radial strain were all significantly lower in patients with SSc than in healthy controls.^15^ Moreover, a recent study^7^ has proved that, while still asymptomatic, GLS worsens over time, suggesting a subtle progression of the cardiac disease which may then become symptomatic.^16^

Nonetheless, GLS has been proved helpful in detecting heart involvement and myocardial fibrosis progression in juvenile SSc, where GLS worsening anticipated the decrease of LV dysfunction.^17^ Given the importance of cardiac involvement on life expectancy in patients with SSc, in this study, we aimed at identifying possible predictors of progression to clinical cardiovascular disease, morbidity and mortality.

## Methods

### Study population

The present prospective longitudinal study enrolled all consecutive patients with a diagnosis of SSc referred by Clinica Medica and other spoke Internal Medicine or Rheumatology Clinics to the Outpatient Cardiology Clinic for Rare Diseases, Marche University Hospital, Ancona, Italy, between February 2016 and February 2022. All patients met the American College of Rheumatology/European League Against Rheumatism classification criteria for SSc.^18^ Exclusion criteria were: structural heart disease, HF with reduced or preserved ejection fraction, moderate or severe valve disease or valve replacement or repair, ischaemic heart disease, and previous episodes of deep vein thrombosis or pulmonary embolism. A complete cardiac examination, including 12-lead electrocardiography (ECG) and echocardiography, was performed in all patients to rule out potential underlying heart conditions before enrolment (see the Data collection section).

The study was carried out according to the Declaration of Helsinki. Informed consent was obtained from all patients. The study was approved by the local ethics committee (Comitato Etico Regionale delle Marche, no. 173/2022). The present manuscript was designed, conducted, and reported according to the STROBE initiative (see Supplementary material online, *Table S1*).^19^

### Data collection

Clinical history and physical examination were collected for all patients at enrolment and during follow-up. At each visit, a complete cardiac examination including a 12-lead ECG was performed by experienced cardiologists.

Patients were stratified in two subsets, limited cutaneous or diffuse cutaneous, based on the extent of skin involvement.

The following clinical characteristics were also collected for all patients: disease duration from the first non-Raynaud’s symptom, autoantibody profile, capillaroscopic pattern, severity of skin induration, presence of other organ systems involvement, previous and ongoing treatment (D.B., C.D., and G.M.). Skin involvement was evaluated by a modified Rodnan Skin Score, which was performed by one experienced assessor (D.B.). Oesophageal involvement was assessed by videofluorography swallow study and high-resolution chest computed tomography (HRCT).^20^ Pulmonary involvement was assessed by HRCT and pulmonary function tests.

Systemic sclerosis disease activity was evaluated using the revised European Scleroderma Trials and Research group activity index (EUSTAR-AI),^21^ and damage accrual was assessed using the Scleroderma Clinical Trials Consortium Damage Index (SCTC-DI).^22^

Patients with a high probability of HF or pulmonary hypertension on echocardiogram were managed according to the current guidelines.

### Echocardiography and speckle-tracking-derived measurements

Detailed methods for echography examination have been previously described.^7,13^ In brief, two experienced operators (G.S. and F.G.) performed all examinations and extracted bidimensional, Doppler and speckle-tracking data. Feasibility of the frame-to-frame tracking technique was obtained by setting the frame rate of digital loops for speckle-tracking analysis between 60 and 80 fps. Global longitudinal strain was derived from specific digital loops obtained by setting the frame rate between 60 and 80 fps using offline software (EchoPAC 13.0; GE Medical Systems, Milwaukee, WI, USA). Only the free-wall segments were used for RV GLS calculations. Strain feasibilities were 96.5% for the LV and 92.6% for the RV. Intra-observer reproducibility was 2.5% and inter-observer reproducibility was 3.1%. Feasibility and reproducibility were comparable to what was already published by our group.^7^

### Outcomes

The primary outcome was the association between left and right GLS and mortality for all causes. The secondary outcome was the association between left and right GLS and hospitalization for cardiovascular causes. Primary endpoint occurrences were documented through the regional database network or direct phone contact with either the patient or their general practitioners up to the end of follow-up (February 2023). A modified Hinkle–Thaler classification was used to categorize deaths into sudden cardiac deaths, non-sudden cardiac deaths (further divided into coronary-related, HF-related, pulmonary hypertension-related, or other), and non-cardiac deaths (further divided into cancer-related, pulmonary-related, or other).^23^ Hospitalizations were defined as a length of stay >12 h in any medical facility and categorized into cardiovascular or non-cardiovascular according to ICD-10 codes at discharge. All endpoints were adjudicated by a committee (G.S., F.G., and A.D.R.), which was also responsible for classifying the type of death and reasons for hospitalization.

### Statistical analysis

Qualitative variables were described as absolute or relative prevalence. Quantitative variables were assessed for normality using the Kolmogorov–Smirnov test and described as mean and standard deviation (SD) or median and first-third quartile, as appropriate. Baseline differences between patients with limited or diffuse involvement were assessed by Fisher’s exact value or χ^2^ analysis for quantitative variables, analysis of variance (ANOVA) for normally distributed quantitative variables, and Kruskal–Wallis ANOVA for non-normally distributed quantitative variables. Missing values were handled by listwise deletion.

Cox regression models were used to test the association between left and right GLS and primary and secondary outcomes. Univariate-derived associations were adjusted by common clinical risk factors, such as age, gender, SSc subset, and left ventricular ejection fraction (LVEF).

If multivariate Cox regression reached statistical significance, a receiver-operating-characteristic (ROC) curve was constructed to find the best cut-off for both left and right GLS and the primary and secondary outcomes. Calculated cut-offs were then used as independent variables to reproduce Kaplan–Meier curves for each primary and secondary endpoint, to provide accurate thresholds for routine use in clinical practice.

Values of *P* < 0.05 (two-tailed) were considered statistically significant. R software (R Foundation for Statistical Computing, Vienna, Austria) and SPSS 25.0 for Windows (SPSS Inc., Chicago, IL, USA) were used for statistical analysis.

## Results

The general characteristics of the population are shown in *Table 1*.

**Table 1:** Baseline characteristics

	SSc (*n* = 164)	Diffuse subset (*n* = 35)	Limited subset (*n* = 129)	*P*-value
Age (years), mean ± SD	58.8 ± 14.0	53.7 ± 16.1	60.2 ± 13.1	0.010
Female gender, *n* (%)	148 (90.8)	31 (88.6)	117 (91.4)	0.607
Hypertension, *n* (%)	57 (35.2)	12 (35.3)	45 (35.2)	0.998
Diabetes mellitus, *n* (%)	8 (4.9)	0 (0.0)	8 (6.3)	0.206
Dyslipidaemia, *n* (%)	35 (21.6)	4 (11.8)	31 (24.2)	0.117
Active smoking, *n* (%)	16 (9.8)	4 (11.4)	12 (9.3)	0.308
Heart rate (b.p.m.), mean ± SD	76.3 ± 14.0	81.6 ± 9.9	75.5 ± 14.4	0.289
BMI (kg/m^2^), mean ± SD	23.5 ± 4.3	21.7 ± 3.4	24.0 ± 4.4	0.006
BNP (pg/mL), median (1st–3rd quartile)	36 (21–273)	55 (19–119)	36 (25–393)	0.472
Creatinine (mg/dL), mean ± SD	0.8 ± 0.3	0.7 ± 0.2	0.8 ± 0.3	0.074
Age at diagnosis (years), mean ± SD	50.6 ± 15.4	44.7 ± 15.7	52.5 ± 15.0	0.011
ANA+, *n* (%)	143 (87.2)	34 (97.1)	109 (84.5)	0.047
Scl70+, *n* (%)	64 (39.0)	23 (65.7)	41 (31.8)	<0.001
ACA+, *n* (%)	57 (34.8)	4 (11.4)	53 (41.1)	0.001
Lung involvement, *n* (%)	98 (59.8)	33 (94.3)	65 (50.4)	<0.001
Skin involvement, *n* (%)	97 (59.1)	31 (88.6)	66 (51.2)	<0.001
Oesophageal involvement, *n* (%)	114 (69.5)	33 (94.3)	81 (62.8)	<0.001
EUSTAR-AI, mean ± SD	1.4 ± 1.6	3.3 ± 1.9	0.8 ± 0.9	<0.001
SCTC-DI, median (1st–3rd quartile)	1 (0–6)	6 (0–8)	0 (0–3)	0.001

ACA, anti-centromere antibodies; ANA, anti-nuclear antibodies; ACE, angiotensin converting enzyme; BMI, body mass index; BNP, brain natriuretic peptide; DLCO, diffusion lung carbon monoxide; EUSTAR-AI, European Scleroderma Trials and Research group activity index; SCTC-DI, Scleroderma Clinical Trials Consortium Damage Index; SD, standard deviation; Scl70, anti-topoisomerase I antibodies; SSc, systemic sclerosis.

The cohort consisted of 164 patients, with a mean age of 58.8 ± 14.0 years, 148 (90.9%) females, followed up for a median of 3.2 years (1st–3rd quartile 1.4–4.8 years). *Table 2* details the main echocardiographic features at enrolment. Patients with the diffuse subset were overall younger and with lower body mass index but, otherwise, showed no significant differences in echocardiographic data when compared with the limited SSc subgroup (*Table 2*).

**Table 2:** Echocardiographic characteristics at enrolment

	SSc (*n* = 164)	Diffuse subset (*n* = 35)	Limited subset (*n* = 129)	*P*-value
LVEDD (mm), mean ± SD	43.8 ± 6.5	44.3 ± 6.9	43.7 ± 6.4	0.643
IV (mm), mean ± SD	9.5 ± 2.2	9.4 ± 2.4	9.5 ± 2.1	0.782
PW (mm), mean ± SD	9.3 ± 2.5	8.8 ± 2.1	9.4 ± 2.6	0.182
iLVEDV (mL/m^2^), mean ± SD	46.1 ± 11.6	49.2 ± 9.6	45.3 ± 12.0	0.239
iLVESV (mL/m^2^), mean ± SD	17.0 ± 6.6	17.8 ± 6.6	16.8 ± 6.6	0.853
LVEF (%), mean ± SD	63.6 ± 7.2	64.7 ± 8.1	63.3 ± 7.0	0.294
iLAV (mL/m^2^), mean ± SD	24.3 ± 9.8	23.2 ± 9.5	24.6 ± 9.8	0.481
iRAV (mL/m^2^), mean ± SD	20.8 ± 9.5	22.9 ± 9.5	20.2 ± 9.5	0.140
*E*/*A*, mean ± SD	1.1 ± 0.4	1.1 ± 0.3	1.1 ± 0.4	0.710
*E*/*e′*, mean ± SD	8.1 ± 3.4	8.2 ± 3.1	8.0 ± 3.5	0.818
RV basal (mm), mean ± SD	34.6 ± 5.3	34.3 ± 5.9	34.7 ± 5.2	0.758
TAPSE (mm), mean ± SD	22.4 ± 3.9	22.4 ± 3.6	22.4 ± 4.0	0.988
FAC (%), mean ± SD	47.3 ± 6.3	44.0 ± 5.4	46.6 ± 7.6	0.519
TR gradient (m/s), mean ± SD	2.5 ± 0.4	2.5 ± 0.4	2.5 ± 0.5	0.900
sPAP (mmHg), mean ± SD	28.9 ± 10.4	29.1 ± 9.0	28.9 ± 11.3	0.733
LV–GLS (%), mean ± SD	−19.7 ± 3.5	−19.4 ± 4.0	−19.8 ± 3.5	0.491
RV–GLS (%), mean ± SD	−20.3 ± 4.6	−19.8 ± 4.9	−20.4 ± 4.3	0.741

FAC, fractional area change; GLS, global longitudinal strain; iLAV, indexed left atrial volume; iLVEDV, indexed left ventricular end-diastolic volume; iLVESV, indexed left ventricular end-systolic volume; iLAV, indexed right atrial volume; iRAV, indexed right atrial volume; IVS, intraventricular septum; LV, left ventricle; LVEDD, left ventricular end-diastolic diameter; LVEF, left ventricular ejection fraction; PW, posterior wall; RV, right ventricle; sPAP, systolic pulmonary artery pressure; TAPSE, tricuspidal antero-posterior systolic excursion; TR, tricuspidal regurgitation.

### Primary outcome

**Figure 1:**
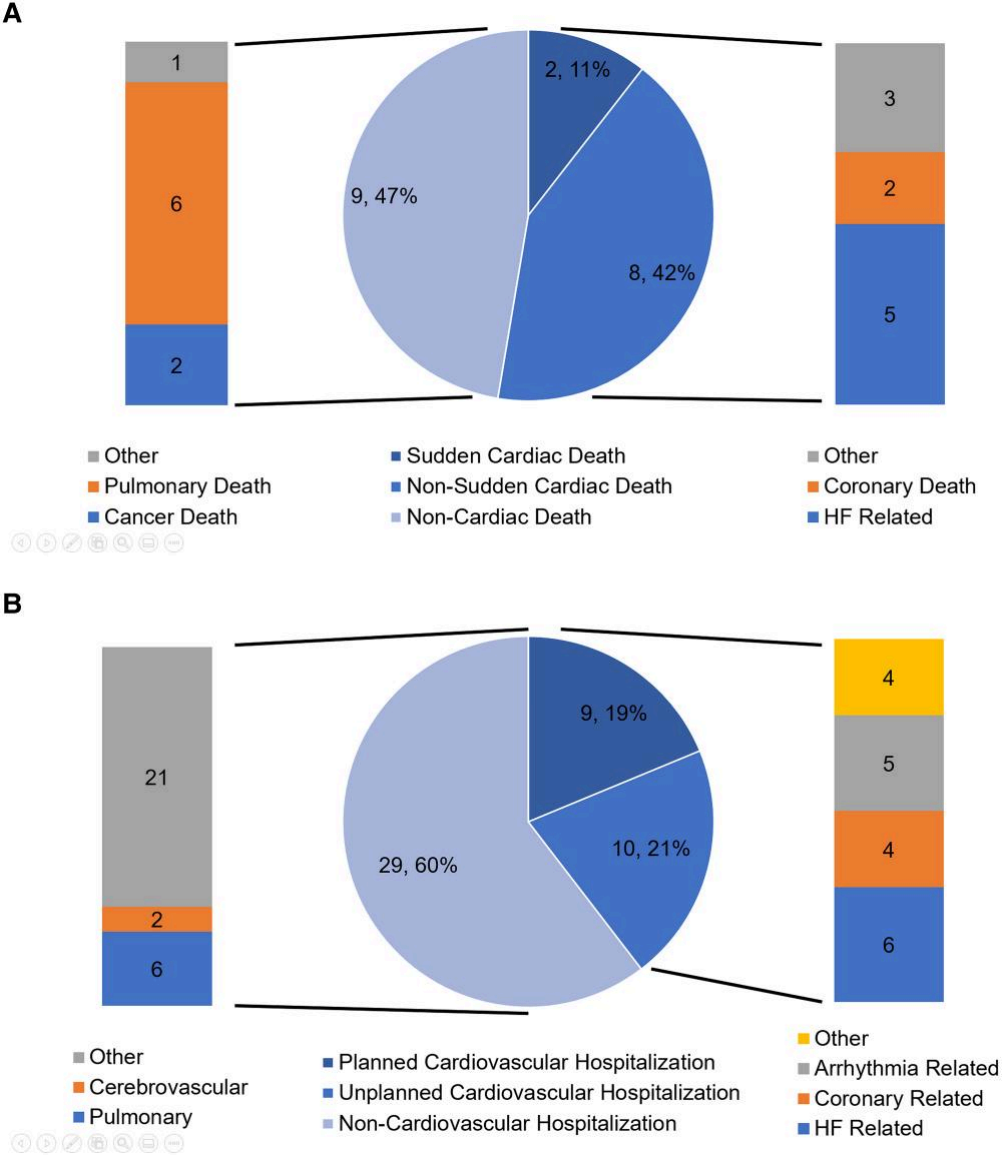
Adjudicated endpoints during follow-up. Panel (*A*) depicts the total number of deaths, divided between cardiovascular and non-cardiovascular, sudden and non-sudden, and by cause. Panel (*B*) shows the total number of hospitalizations, divided between cardiovascular and non-cardiovascular, planned or unplanned, and by cause. HF, heart failure.

Out of 164 patients, 19 (11.5%) died during follow-up and 48 (29.3%) were hospitalized for cardiovascular reasons. *Figure 1* details causes of death and cardiovascular hospitalizations, as adjudicated by the committee.

**Figure 2:**
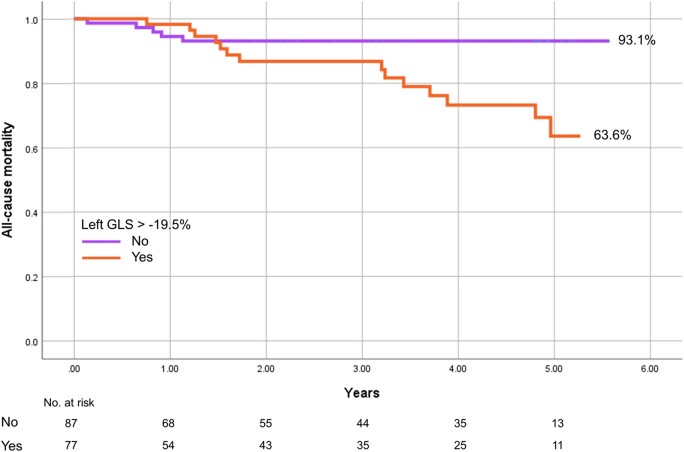
Time to all-cause death, according to left ventricle global longitudinal strain. Kaplan–Meier curves show time to all-cause death, according to the left global longitudinal strain cut-off. GLS, global longitudinal strain.

Left GLS at enrolment was associated with an increased risk of death for all causes [hazard ratio (HR) 1.19; 95% confidence interval (CI) 1.05–1.35; *P* = 0.007]. Furthermore, the association remained significant after adjustment for age, gender, SSc subset, LVEF, disease activity (EUSTAR), and SCTC damage index (HR 1.15; 95% CI 1.02–1.28; *P* = 0.036; *Table 3A*). A cut-off value of −19.5% was selected as the most accurate in predicting death (sensitivity 72%, specificity 62%; area under the curve [AUC] 0.645). Patients with left GLS worse (i.e. higher) than −19.5% had a 6.3-fold increased risk of death over the follow-up period (6.9 vs. 36.4%; *P* = 0.012; *Figure 2*).

**Table 3:** Logistic regression model for all-cause mortality

A. Left global longitudinal strain								
	Univariable				Multivariable			
Variable	OR	95% CI lower bound	95% CI upper bound	*P*-value	OR	95% CI lower bound	95% CI upper bound	*P*-value
Left GLS (for each 1%)	**1**.**19**	**1**.**05**	**1**.**35**	**0.007**	**1**.**15**	**1**.**02**	**1**.**28**	**0.036**
Age (for each year)	**1**.**08**	**1**.**03**	**1**.**13**	**<0**.**001**	**1**.**07**	**1**.**01**	**1**.**13**	**0.032**
Male gender	1.77	0.51	6.11	0.363	2.67	0.68	10.41	0.167
Diffuse SSc subset	1.11	0.42	2.93	0.836	1.25	0.46	3.45	0.659
LVEF (for each 1%)	0.978	0.92	1.03	0.436	1.03	0.95	1.11	0.457
EUSTAR-AI (for each point)	1.06	0.80	1.34	0.692	1.15	0.60	2.18	0.680
SCTC-DI (for each point)	**1**.**14**	**1**.**03**	**1**.**27**	0.012	**1**.**14**	**1**.**02**	**1**.**28**	**0.035**

B. Right global longitudinal strain								
	Univariable				Multivariable			
Variable	OR	95% CI lower bound	95% CI upper bound	*P*-value	OR	95% CI lower bound	95% CI upper bound	*P*-value
Right GLS (for each 1%)	**1**.**08**	**1**.**02**	**1**.**14**	**0.009**	**1**.**04**	**1**.**02**	**1**.**14**	**0.041**
Age (for each year)	**1**.**08**	**1**.**03**	**1**.**13**	**<0**.**001**	**1**.**10**	**1**.**04**	**1**.**17**	**0.001**
Male gender	1.77	0.513	6.11	0.363	3.51	0.57	11.59	0.173
Diffuse SSc subset	1.11	0.42	2.93	0.836	0.80	0.16	4.06	0.790
TAPSE (for each mm)	0.90	0.81	1.21	0.710	1.04	0.90	1.20	0.586
EUSTAR-AI (for each point)	1.06	0.80	1.34	0.692	0.78	0.46	1.32	0.364
SCTC-DI (for each point)	**1**.**14**	**1**.**03**	**1**.**27**	**0.012**	**1**.**22**	**1**.**06**	**1**.**40**	**0.004**

CI, confidence interval; EUSTAR-AI, European Scleroderma Trials and Research group activity index; GLS, global longitudinal strain; OR, odds ratio; SCTC-DI, Scleroderma Clinical Trials Consortium Damage Index; TAPSE, tricuspid annulus plane systolic excursion; LVEF, left ventricular ejection fraction.

Bold values indicates *P* < 0.05.

**Figure 3:**
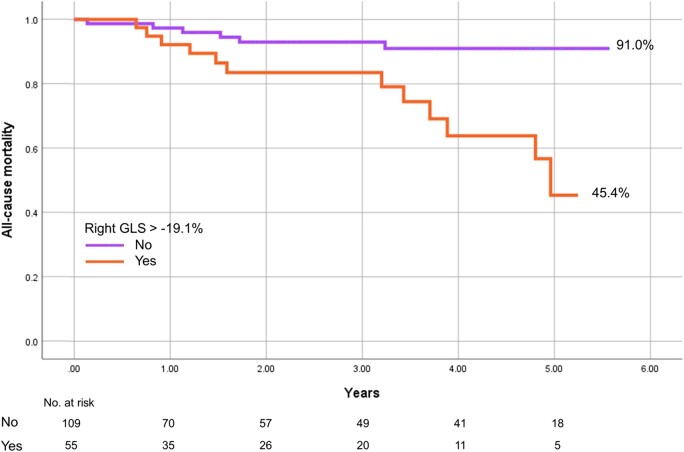
Time to all-cause death, according to right ventricle global longitudinal strain. Kaplan–Meier curves show time to all-cause death, according to the right global longitudinal strain cut-off. GLS, global longitudinal strain.

Right GLS at enrolment presented a significant association with the risk of death for all causes (HR 1.08; 95% CI 1.02–1.14; *P* = 0.009), even when adjusted for age, gender, SSc subset, tricuspidal antero-posterior systolic excursion (TAPSE), disease activity (EUSTAR), and SCTC damage index (HR 1.04; 95% CI 1.02–1.14; *P* = 0.041; *Table 3B*). A cut-off value of −19.1% was selected as the most accurate in predicting death (sensitivity 72%, specificity 70%; AUC 0.695). Patients with right GLS worse (i.e. higher) than −19.1% had a 6.1-fold increased risk of death over the follow-up period (9.0 vs. 54.6%; *P* = 0.001; *Figure 3*).

**Figure 4:**
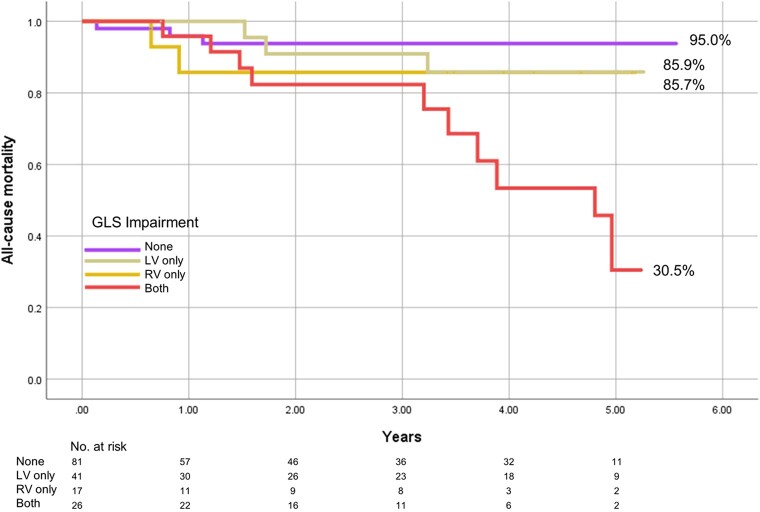
Time to all-cause death, according to biventricular global longitudinal strain. Kaplan–Meier curves show time to all-cause death, according to unilateral or bilateral global longitudinal strain impairment. GLS, global longitudinal strain.

Both LV and RV GLSs performed much better as predicting the primary endpoint than traditional markers of systolic function such as LVEF (AUC 0.515), TAPSE (AUC 0.547), or systolic pulmonary artery pressure (sPAP; AUC 0.598). According to the cut-offs defined above, an impairment of both LV and RV GLSs seems to confer the higher risk of all-cause death (69.5%; *Figure 4*). Patients with biventricular GLS impairment had a 4.2-fold increased risk of death when compared with patients with only LV impairment (14.3%), 4.9-fold increased risk when compared with only RV impairment (14.1%), and 13.9-fold increased risk when compared with normal biventricular GLS (5%; *P* < 0.001).

### Secondary outcome

**Figure 5:**
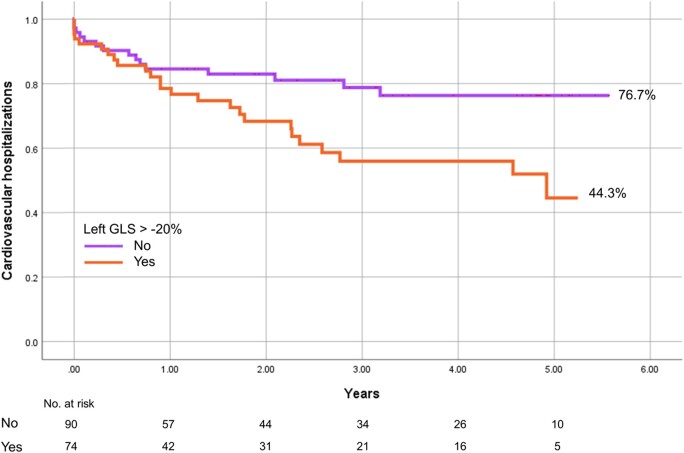
Time to the first hospitalization, according to left global longitudinal strain. Kaplan–Meier curves show the time to the first hospitalization, according to the left global longitudinal strain cut-off. GLS, global longitudinal strain.

At Cox univariate regression analysis, left GLS at enrolment was associated with an increased incidence of hospitalizations (HR 1.15; 95% CI 1.02–1.31; *P* = 0.036). The association was confirmed when adjusted for SSc subset, gender, age, LVEF, disease activity (EUSTAR), and SCTC damage index (HR 1.11; 95% CI 1.01–1.22; *P* = 0.048; *Table 4A*). A cut-off value of −20% was selected as the most accurate in predicting hospitalizations (sensitivity 72%, specificity 55%; AUC 0.634). Patients with left GLS worse (i.e. higher) than −20% had a 2.5-fold increased risk of hospitalization over the follow-up period (23.3 vs. 55.7%; *P* = 0.013; *Figure 5*).

**Table 4:** Logistic regression model for cardiovascular hospitalizations

A. Left global longitudinal strain								
	Univariable				Multivariable			
Variable	OR	95% CI lower bound	95% CI upper bound	*P*-value	OR	95% CI lower bound	95% CI upper bound	*P*-value
Left GLS (for each 1%)	**1**.**15**	**1**.**02**	**1**.**31**	**0.036**	**1**.**11**	**1**.**01**	**1**.**22**	**0.048**
Age (for each year)	**1**.**07**	**1**.**02**	**1**.**13**	**0.003**	1.02	0.99	1.05	0.243
Male gender	0.71	0.21	5.33	0.736	0.96	0.28	3.31	0.959
Diffuse SSc subset	2.01	0.69	5.19	0.144	1.21	0.41	3.53	0.731
LVEF (for each 1%)	0.98	0.92	1.05	0.578	1.03	0.98	1.09	0.309
EUSTAR-AI (for each point)	0.86	0.57	1.28	0.454	1.06	0.76	1.46	0.748
SCTC-DI (for each point)	**1**.**11**	**1**.**00**	**1**.**23**	**0.046**	1.05	0.97	1.14	0.203

B. Right global longitudinal strain								
	Univariable				Multivariable			
Variable	OR	95% CI lower bound	95% CI upper bound	*P*-value	OR	95% CI lower bound	95% CI upper bound	*P*-value
Right GLS (for each 1%)	**1**.**12**	**1**.**04**	**1**.**21**	**0.002**	**1**.**05**	**1**.**00**	**1**.**10**	**0.049**
Age (for each year)	**1**.**07**	**1**.**02**	**1**.**13**	**0.003**	**1**.**04**	**1**.**01**	**1**.**07**	**0.013**
Male gender	0.71	0.21	5.33	0.736	0.70	0.15	3.33	0.668
Diffuse SSc subset	2.01	0.69	5.19	0.144	1.23	0.37	4.09	0.747
TAPSE (for each mm)	0.90	0.79	1.01	0.066	1.06	0.94	1.19	0.355
EUSTAR-AI (for each point)	0.86	0.57	1.28	0.454	1.04	0.75	1.42	0.821
SCTC-DI (for each point)	**1**.**11**	**1**.**00**	**1**.**23**	**0.046**	1.09	0.99	1.20	0.089

CI, confidence interval; EUSTAR-AI, European Scleroderma Trials and Research group activity index; GLS, global longitudinal strain; OR, odds ratio; SCTC-DI, Scleroderma Clinical Trials Consortium Damage Index; TAPSE, tricuspid annulus plane systolic excursion; LVEF, left ventricular ejection fraction.

Bold values indicates *P* < 0.05.

**Figure 6.**
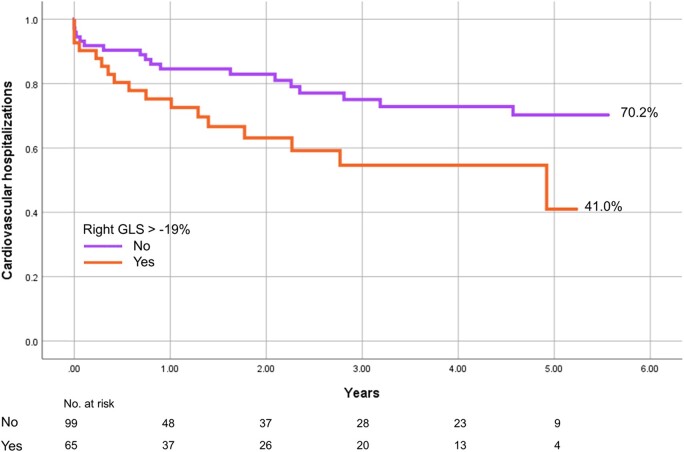
Time to the first hospitalization, according to the right global longitudinal strain. Kaplan–Meier curves show the time to the first hospitalization, according to the right global longitudinal strain cut-off. GLS, global longitudinal strain.

Right GLS at enrolment presented a significant association with an increased risk of hospitalization (HR 1.12; 95% CI 1.04–1.21; *P* = 0.002). The association was confirmed when adjusted for age, gender, SSc subset, TAPSE, disease activity (EUSTAR), and SCTC damage index (HR 1.05; 95% CI 1.00–1.10; *P* = 0.049; *Table 4B*). A cut-off value of −19.0% was selected as the most accurate in predicting hospitalization (sensitivity 77%, specificity 62%; AUC 0.752). Patients with right GLS worse (i.e. higher) than −19.0% had a two-fold increased risk of hospitalization over the follow-up period (29.8 vs. 59.0%; *P* = 0.035; *Figure 6*).

Similarly to the primary endpoint, both LV and RV GLSs performed better than traditional markers of systolic function such as LVEF (AUC 0.567), TAPSE (AUC 0.541), or sPAP (AUC 0.623).

**Figure 7.**
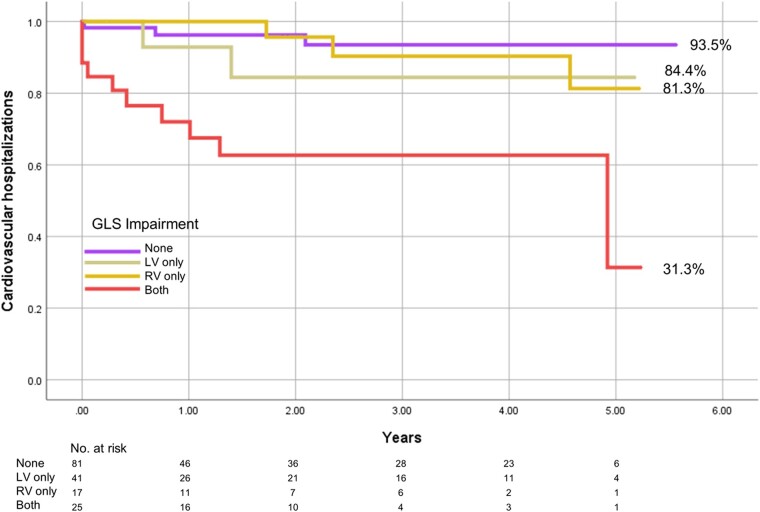
Time to first hospitalization, according to biventricular global longitudinal strain. Kaplan–Meier curves show time to all-cause death, according to unilateral or bilateral global longitudinal strain impairment. GLS: global longitudinal strain.

According to the cut-offs defined above, an impairment of both LV and RV-GLS seems to confer the higher risk of all cardiovascular hospitalization (68.7%; *Figure 7*). Patients with biventricular GLS impairment had a 4.4-fold increased risk of death when compared with patients with only LV impairment (15.6%), 3.7-fold increased risk when compared with only RV impairment (18.7%), and 9.2-fold increased risk when compared with normal biventricular GLS (7.5%; *P* < 0.001).

## Discussion

Cardiac involvement, although often asymptomatic,^24^ is an important manifestation of SSc and is responsible for much of SSc-related morbidity and mortality.^25^ While the first papers on SSc focused mainly on right heart disease and pulmonary arterial hypertension, a frequent and global involvement of both ventricles has soon been pointed out, with left ventricular damage as the main target of contemporary cardiac assessment of all patients with SSc.^26^

Allanore and Meune^4^ had already described the primary heart involvement in SSc, suggesting a microvascular origin leading to myocardial fibrosis. In our previous research,^7,13^ we added evidence regarding SSc-related cardiomyopathy as a frequent, subclinical, and progressive disease and confirmed the hypothesis of a microvascular involvement employing speckle-tracking GLS.

Later on, Bruni and Ross^25^ highlighted the challenges related to identifying primary heart involvement in SSc, albeit the high prevalence of histological findings at autoptic reports and suggested a systematic approach to evaluate SSc cardiomyopathy.

Speckle-tracking GLS is non-invasive, cost-effective, and reproducible, but it is seldom applied in clinical practice, with the only notable exception being the cardio-oncology field, in which the use of this technique as a predictor of ventricular dysfunction is recommended by the European guidelines.

A recent metanalysis by Qiao et al.,^15^ including 31 case–control studies published between 2011 and 2022 confirms that both RV and LV GLS is significantly lower in patients affected by SSc compared to healthy controls, thus further supporting the value of speckle-tracking-derived GLS as a diagnostic tool in SSc.

Nonetheless, numerous research works^27–29^ on both cardiac and systemic diseases have also described the usefulness of GLS as a predictor of morbidity and mortality.

In the current study, we wanted to analyse how clinical outcomes and prognosis are affected in patients with SSc and no other overt cardiac disease. We were able to identify both right and left ventricular GLS as predictors of all-cause mortality and hospitalizations. Specific optimal cut-off values, assessed through ROC curves, were found to obtain a good sensitivity as well as a positive predictive value for the GLS technique. All values found ranged between −19 and −20% for all our study outcomes, indicating this as a sweet spot for predicting clinical complications in patients with SSc.

From our data, RV-GLS (assessed as RV free-wall strain) outperformed LV GLS in terms of sensitivity and specificity for both primary and secondary endpoints, while an impaired LV-GLS was associated with a larger risk of all-cause death or cardiovascular events. Moreover, having a biventricular impairment at enrolment largely increases the risk of both all-cause mortality (*Figure 3*) and cardiovascular hospitalizations (*Figure 6*) even when compared with only one (either LV or RV) impaired GLS. These results underline the need for a comprehensive assessment of both ventricular chambers in patients with SSc and the incremental prognostic information provided by both LV and RV GLS. The synergistic effect of LV and RV altered deformation also adds up to the clinical data pointing at a global involvement of the heart in SSc pathophysiology.^3,14^

On another note, several studies^10,30,31^ have described the use of cardiac magnetic resonance as helpful in identifying patients at higher risk of morbidity and mortality in SSc. Diffuse myocardial fibrosis, as assessed by native T1 and extracellular volume, can be found already in patients with very early diagnosis of SSc, preceding subclinical functional myocardial impairment by GLS and predicting all-cause and cardiovascular mortality.^5,11^ However, such a tool is limited by its availability and its impact on cost and time when compared to speckle-tracking echocardiography, which has recently risen as a promising tool due to its ability to non-invasively and quickly detect SSc-related cardiomyopathy in its subclinical phase.^14,15^

We, therefore, suggest that when assessing patients with SSc, a thorough cardiac evaluation and follow-up should be implemented, including echocardiographic assessment both at baseline and following visits. Biventricular GLS (using free-wall segments for RV GLS) should be calculated and used to screen high-risk patients.

To date, there is no specific treatment for heart involvement in SSc. However, therapeutic strategies aimed at reducing myocardial fibrosis and inflammation are emerging in the form of tocilizumab (an IgG1 subclass humanized anti-human interleukin-6 receptor antibody)^32^ and interleukin-1 inhibitors.^33^

Early identification of high-risk patients may guide the use of approved and future pharmacological strategies potentially damaging or improving cardiac function in SSc.^21,34^

### Study limitations

Our paper presents some limitations that should be elucidated. First, despite being one of the largest datasets currently available on heart involvement in SSc, the raw number of events does not permit to study subgroups nor discriminate between cardiovascular and non-cardiovascular death as the main drive associated with a lower GLS. Moreover, the speckle-tracking analysis, despite surely becoming increasingly common in the last decade, still suffers from suboptimal feasibility, especially for the right ventricle. Therefore, it would be unwise to generalize our findings in patients whose acoustic windows are not good enough to perform high-quality examinations, or who are suffering from irregular arrhythmias (mainly atrial fibrillation) that would offset the software calculations. Lastly, no consensus exists on the proper optimal management of patients with impaired GLS but no overt cardiac disease (diagnostic pathway/treatment).

## Conclusions

Right and left ventricular GLS is an accurate predictor of all-cause mortality and hospitalizations over a 3-year follow-up. Our data add evidence to the notion that a complete cardiac evaluation should be implemented in all patients with SSc, including biventricular GLS assessment both at baseline and during following visits. Early identification of high-risk patients through GLS may justify different follow-up protocols and treatment strategies.

## Supplementary Material

oeae023_Supplementary_Data

## Data Availability

The data underlying this article will be shared on reasonable request to the corresponding author.

## Lead author biography

Dr Giulia Stronati graduated from the Polytechnic University of Marche in 2016, after working for a year at the NHS Buckinghamshire Trust in the UK, she is currently a cardiology and research fellow at the Cardiology and Arrhythmology Clinic of the Marche University Hospital in Ancona, Italy. She is a PhD fellow in Biomedical Sciences and her main areas of interest include cardiovascular imaging, pulmonary hypertension, cardiomyopathies, and emergency cardiology.
